# Postoperative bioactive adrenomedullin is associated with the onset of ARDS and adverse outcomes in patients undergoing open thoracoabdominal aortic surgery

**DOI:** 10.1038/s41598-024-63412-1

**Published:** 2024-06-04

**Authors:** Panagiotis Doukas, Oliver Hartmann, Jelle Frankort, Birte Arlt, Hanif Krabbe, Michael Johan Jacobs, Andreas Greiner, Jan Paul Frese, Alexander Gombert

**Affiliations:** 1 Department of Vascular Surgery, European Vascular Center Aachen-Maastricht, RWTH University Hospital Aachen, 52074 Aachen, Germany; 2 SphingoTec GmbH, Hennigsdorf, Berlin, Germany; 3 Department of Vascular Surgery, Charité—Universitätsmedizin Berlin, Berlin, Germany

**Keywords:** Biomarkers, Diagnostic markers, Predictive markers, Prognostic markers, Medical research, Outcomes research, Aneurysm, Aortic diseases

## Abstract

Cytokine-mediated systemic inflammation after open thoracoabdominal aortic aneurysm (TAAA) repairs plays a pivotal role in disrupting circulatory homeostasis, potentially leading to organ dysfunction. The bioactive form of adrenomedullin (bio-ADM) is a peptide hormone with immunomodulatory and vasomotor effects, making it a potential diagnostic agent in these cases. This retrospective, bicentric study, conducted between January 2019 and December 2022, recruited 36 elective open TAAA repair patients in two German centres. Serum and plasma samples were collected at multiple time points to measure bio-ADM levels. The primary objective was to evaluate the association of bio-ADM levels with the onset of acute respiratory distress syndrome (ARDS), with secondary endpoints focusing on mortality and SIRS-related morbidity. Results showed a significant association between postoperative bio-ADM levels (12–48 h after surgery) and the onset of ARDS (*p* < .001), prolonged ventilation (*p* = .015 at 12h after surgery), atrial fibrillation (*p* < .001), and mortality (*p* = .05 at 24h). The biomarker was also strongly associated with sepsis (*p* = .01 at 12 h) and multi-organ dysfunction syndrome (MODS) (*p* = .02 at 24 h after surgery). The study underscores the potential utility of bio-ADM as a diagnostic tool for identifying patients at risk of postoperative complications following open TAAA repairs.

## Introduction

Postoperative hemodynamic instability and organ dysfunction are significant complications following open thoracoabdominal aortic aneurysm (TAAA) repairs and can have a detrimental impact on the patient’s postoperative course and outcome^1^. Cytokine-mediated systemic inflammation plays a crucial role in the underlying mechanisms that disrupt the patient's circulatory homeostasis. This inflammation compromises the endothelial barrier, reduces peripheral vasotonus, and causes a shift of fluid from the intraluminal space to the interstitium, potentially leading to organ dysfunction^2^. Both pathogens and non-infectious stimuli, such as major surgery, can exacerbate this cytokine surge and worsen these effects^2^.

Patients undergoing open TAAA repair are at an increased risk of developing systemic inflammatory response syndrome (SIRS)^3^. The surgical trauma resulting from thoraco-laparotomy to gain access to the thoracoabdominal aorta^4^ and the use of extracorporeal circulation^5,6^ have been found to induce cytokine expression. Additionally, intestinal ischemia–reperfusion injury following supracoeliac aortic cross clamping can further enhance cytokine induction and cellular stress^7,8^. The respiratory system is often involved in the systematic inflammation cascade^9^. Given the heightened risk of SIRS and its association with significant morbidity and mortality rates, postoperative monitoring and timely management of at-risk patients are crucial. Monitoring the integrity of the endothelial barrier and vascular tone are central components of this effort^10^.

Adrenomedullin (ADM) is a peptide hormone composed of 52 amino acids^11^ that was initially discovered in human pheochromocytoma tissue^12^. It plays a vital role in regulating the endothelial barrier, peripheral resistance, and exhibits immunomodulatory effects^13^. ADM receptors are present on various cell types, including endothelial cells, vascular smooth muscle cells (VSMC), cardiomyocytes, and macrophages^13^. The bioactive form of ADM (bio-ADM) has a dual circulatory effects: inducing peripheral vasodilation by binding to its receptors on endothelial cells and VSMC^14^, and simultaneously tightening and stabilizing the endothelial barrier^13^. Although these effects may appear contradictory, bio-ADM holds promise as a diagnostic marker for SIRS and sepsis and potentially as a target for therapeutic agents^13–15^. Increased levels of bio-ADM are associated with higher mortality in septic patients^16–19^ and have been proposed as a sepsis biomarker independent of morbidity^20^. In a previous study by our group, we investigated the role of bio-ADM during the postoperative phase of patients undergoing open or endovascular aortic reconstructions and found a significant association between bio-ADM levels and the development of cardiogenic shock or fatal outcomes^21^.

Building upon the findings of our previous work, the objective of this bicentric study is to explore the potential diagnostic capability of bio-ADM for ARDS, death and SIRS-related complications, such as sepsis, in a distinct group of patients undergoing open TAAA repairs.

## Methods

### Study design

In this retrospective, observational trial, the medical records of 36 patients planned for elective open TAAA in two German centres (University Hospital Aachen and Charité–University Hospital Berlin) from January 2019 to December 2022 were evaluated. Exclusion criteria were age < 18 years and emergent cases. The study was designed in accordance with the STROBE criteria and the Declaration of Helsinki and it was approved by the Ethics Committees of the participating centres (EK010/19). Written informed consent was obtained from all patients prior to enrolment. The details of patient recruitment and material acquisition were preregistered as part of a wider research project at clinicaltrials.gov (NCT04087161).

### Surgery and postoperative care

The surgical protocol for open reconstructions of the thoracoabdominal aorta has been previously described in detail^22^ and it included retrograde distal aortic perfusion through extracorporeal circulation with left femoro-femoral cannulation and selective perfusion of the visceral arteries. During thoraco-laparotomy, one-lung ventilation of the right lung for at least the thoracic part of the procedure, including the aortic preparation and the proximal aortic anastomosis-suture, as well as the right-sided positioning on a beanbag were part of the standard procedure for aortic surgery in both centres. The renal arteries were infused with one litre of 4°C Custodiol® (Dr. Franz Köhler Chemie, Bensheim, Germany) each. During the surgery, every patient underwent cerebrospinal fluid drainage and intraoperative monitoring of motor-evoked potentials to evaluate the spinal cord's functional integrity. Additionally, a mild systemic hypothermia approach was applied maintaining a permissive temperature of 33 °C.

After surgery, patients were closely monitored in the intensive care unit. To ensure adequate spinal cord perfusion, mean arterial pressure (MAP) was meticulously maintained above 80 mmHg. This was achieved by adjusting the administration of catecholamines, mainly norepinephrine, to meet the MAP target. From the second postoperative day, sedation levels were gradually reduced to encourage spontaneous breathing. If patients could breathe on their own without difficulty and displayed appropriate neurological responses, extubation was considered^23^. To assist in the complete expansion of the lungs—especially the left lung, which had collapsed during surgery—intermittent non-invasive ventilation was used to prevent respiratory fatigue. In instances of respiratory decline, re-intubation was contemplated, and for patients requiring extended mechanical ventilation, tracheostomy was evaluated as an option.

### Material acquisition

Serum and plasma samples were collected from each patient at five specific timepoints: before the surgery (baseline), immediately after the surgery, and at 12-, 24- and 48-h post-surgery. Following collection, the blood samples were subjected to centrifugation at 3000 rotations per minute for 10 min, and the resulting supernatants were stored at a temperature of -80°C. The concentration of bio-ADM in the EDTA plasma was determined using the immunoluminometric assay sphingotest® bio-ADM® (SphingoTec GmbH, Hennigsdorf, Germany), following the previously described methodology^24^. The assay and the subsequent analysis were experimental and the kit was used as a “Research Use Only” (RUO) device. The laboratory conducting the biomarker analysis remained unaware of the patients' clinical and demographic information. Based on the manufacturer’s instruction for use, the 97.5th percentile for sphingotest® bio-ADM® in healthy adult subjects is 29 pg/mL (90% CI 27–38 pg/mL). The clinical cut-off for patients with sepsis and septic shock is 70 pg/mL^16,19^.

### Endpoints

The primary endpoint of this retrospective analysis was the evaluation of respiratory complications after open TAAA repairs—particularly the occurrence of ARDS and prolonged ventilation. The diagnosis of acute respiratory distress syndrome (ARDS) was made based on the Berlin definition. ARDS was classified into three categories, namely mild (ARDS 1), moderate (ARDS 2), and severe (ARDS 3), depending on the degree of arterial hypoxemia, as revealed from the calculated Horowitz-index^25^. Prolonged ventilation was defined as mechanical ventilation for a duration of more than 21 days^26^. Secondarily, we investigated the postoperative complications associated with SIRS and postoperative mortality. Patients were classified as septic if there was suspicion of infection, and their sequential organ failure assessment (SOFA) score demonstrated an increase of at least 2 points^27^. The diagnosis of multi-organ dysfunction syndrome (MODS) was made in patients who experienced failure in two or more vital organ systems^28^. Mortality was defined as a fatal outcome after surgery.

### Statistics

Continuous variables were presented as median [interquartile range]; categorical variables were reported as absolute frequencies (n) and percentages. Group comparisons of continuous variables were performed using the Kruskal–Wallis test, and categorical data were compared using Pearson's Chi-squared Test for Count Data. Statistical significance was determined at p < 0.05, with a 95% confidence interval (CI) and given the exploratory nature of this analysis no adjustment for multiple testing was made. The predictive accuracy of bio-ADM in anticipating the onset of various complications was evaluated by generating a Receiver Operating Characteristic (ROC) curve and assessing the concordance index (c-index). Optimal cut points were determined based on the Youden index, and sensitivity and specificity calculated for illustration. The statistical analyses were performed using R version 4.2.2 (http://www.r-project.org, with libraries rms, Hmisc, ROCR) and Statistical Package for the Social Sciences (SPSS) version 22.0 (SPSS Inc., Chicago, Illinois, USA).

## Results

Out of the 36 patients enrolled in this study, the median [IQR] age was 56 [43–63] years (Table 1). Among the participants, 69.4% were men, and the median [IQR] maximum aortic diameter was 6.1 [5.6–6.7] cm. The distribution of aortic repairs based on the Crawford classification revealed that type 2 repairs were performed in 38.2% of cases, followed by type 3 repairs in 29.4% of cases, and type 4 repairs in 20.6% of cases. Nine patients died postoperatively (25%). The onset of multi-organ dysfunction syndrome postoperatively was significantly associated with mortality (8.3% vs. 75%, *p* = 0.001).

**Table 1:** Demographic details, comorbidities and laboratory parameters at baseline and their association with mortality, sepsis, onset of ARDS and bio-ADM levels at 24 h after surgery. The median of bio-ADM at 24 h postoperatively (39.7 pg/mL) was used to dichotomize the continuous variable.

Demographic details and comorbidities	n	All	Non-fatal	Fatal	*p*-value	No sepsis	Sepsis	*p*-value	No ARDS	ARDS	*p*-value	Bio-ADM, 24 h [0,39.7) pg/mL	Bio-ADM, 24 h (39.7,180] pg/mL	*p*-value
Age (years)—median [IQR]	36	56 [42.75–63.25]	53 [42.25–60.75]	62 [58–65]	.15	53.5 [43.25–63.25]	58 [39–62.5]	.92	49.5 [45.25–58.25]	58.5 [42.25–64.75]	.19	54 [41.75–63.25]	57 [43–63]	.69
Men (%)	36	25 (69.4)	21 (80.8)	3 (33.3)	.03*	15 (68.2)	7 (63.6)	1	9 (75)	14 (63.6)	.77	10 (55.6)	13 (76.5)	.34
Obesity (%)	36	3 (8.3)	2 (7.7)	1 (11.1)	1	1 (4.5)	2 (18.2)	.52	0 (0)	3 (13.6)	.48	0 (0)	3 (17.6)	.21
BMI (kg/m2)—median [IQR]	36	23 [20.85–25.77]	23.65 [22.2–26.45]	20.9 [16.9–22.6]	.07	23.65 [21.08–26.45]	22.6 [18.9–25]	.43	23.1 [21.88–25.02]	22.95 [19.65–25.92]	.84	23.1 [22.2–25.6]	24.3 [22.3–25.7]	.6
Hypertension (%)	36	28 (77.8)	19 (73.1)	8 (88.9)	.61	15 (68.2)	10 (90.9)	.31	8 (66.7)	18 (81.8)	.57	12 (66.7)	15 (88.2)	.26
Diabetes mellitus type II (%)	36	3 (8.3)	1 (3.8)	2 (22.2)	.31	2 (9.1)	1 (9.1)	1	2 (16.7)	1 (4.5)	.58	0 (0)	2 (11.8)	.44
COPD (%)	35	9 (25.7)	5 (20)	4 (44.4)	.32	6 (27.3)	3 (27.3)	1	3 (25)	6 (28.6)	1	4 (22.2)	4 (25)	1
Previous aortic surgery of any kind (%)	36	24 (66.7)	17 (65.4)	6 (66.7)	1	13 (59.1)	8 (72.7)	.7	7 (58.3)	15 (68.2)	.84	9 (50)	13 (76.5)	.2
Aorto-bifemoral graft in history (%)	36	11 (30.6)	8 (30.8)	3 (33.3)	1	9 (40.9)	2 (18.2)	.36	5 (41.7)	6 (27.3)	.64	6 (33.3)	5 (29.4)	1
Max. aortic diameter (cm)—median [IQR]	32	6.1 [5.57–6.73]	6 [5.43–6.2]	6.7 [6–7.2]	.12	6 [5.5–6.6]	6.2 [5.55–6.8]	.59	6.3 [6–6.72]	6 [5.5–6.7]	.46	6 [4.85–6.3]	6.2 [6–6.9]	.08
Rupture (%)	36	6 (16.7)	2 (7.7)	4 (44.4)	.04*	3 (13.6)	3 (27.3)	.63	3 (25)	3 (13.6)	.72	2 (11.1)	3 (17.6)	.95
Crawford Type (%)	33				.288			.451			.316			.328
1		3 (8.8)	3 (13)	0 (0)		1 (5.6)	0 (0)		1 (10)	1 (5)		2 (11.8)	1 (6.7)	
2		13 (38.2)	7 (30.4)	4 (50)		5 (27.8)	6 (54.5)		3 (30)	8 (40)		5 (29.4)	7 (46.7)	
3		10 (29.4)	8 (34.8)	2 (25)		6 (33.3)	4 (36.4)		2 (20)	8 (40)		4 (23.5)	6 (40)	
4		7 (20.6)	5 (21.7)	1 (12.5)		5 (27.8)	1 (9.1)		4 (40)	2 (10)		5 (29.4)	1 (6.7)	
Laboratory parameters at baseline														
GFR (ml/min/1.73m^2^)—median [IQR]	34	83.5 [58.6–97.57]	91.1 [67–104.57]	56.7 [39–83]	.03*	85.25 [61.75–97.1]	67.9 [47.85–100.95]	.57	85.75 [57.52–108.2]	83 [61–96.1]	.43	84 [56.7–97.5]	77.2 [63.25–99.55]	1
Creatinine (mg/dl)—median [IQR]	34	.96 [.76–1.23]	0.94 [0.74–1.13]	1.08 [0.78–1.81]	.26	0.87 [0.74–1.11]	1.08 [0.86–1.52]	.3	0.91 [0.77–1.1]	0.95 [0.75–1.23]	.64	0.85 [0.74–1.09]	0.96 [0.78–1.23]	.38
Leukocytes (/nl)—median [IQR]	34	8 [6.4–9.1]	7.55 [5.5–8.47]	9.5 [9–13.1]	.01*	7.55 [5.3–9.1]	8.7 [8–9.25]	.12	7.75 [6.8–9.35]	8.3 [6.3–9.1]	.87	7.5 [5.2–9]	8.05 [6.77–8.88]	.44
CRP (mg/l)—median [IQR]	32	7.2 [2.18–25.25]	6.8 [2.48–17.5]	14.4 [2.03–160.2]	.53	7.9 [2.2–26.9]	7 [2.75–18.45]	.94	9.35 [6.38–50.92]	3.25 [1.8–16.77]	.07	4.5 [1.9–8.5]	7.05 [2.43–23.97]	.38
PCT (ng/ml)—median [IQR]	13	.05 [.03-.17]	0.04 [0.02–0.05]	0.13 [0.09–0.44]	.09	0.04 [0.02–0.53]	0.09 [0.08–0.13]	.31	0.04 [0.02–0.35]	0.09 [0.08–0.17]	.3	0.03 [0.02–0.05]	0.09 [0.08–0.17]	.03*
Thrombocytes (/nl)—median [IQR]	34	249 [204.75–284.75]	249 [210.75–288.75]	254 [203–286]	.82	250.5 [210.25–304.25]	242 [208.5–276]	.73	252.5 [232–375.75]	242 [198–266]	.16	251 [226–286]	216 [196.75–263.75]	.2

ARDS, acute respiratory distress syndrome; BMI, body-mass index; COPD, chronic obstructive pulmonary disease; GFR, glomerular filtration rate; CRP, C-reactive protein; PCT, procalcitonin.

### Postoperative bio-ADM levels are associated with ARDS severity and prolonged ventilation.

**Figure 1:**
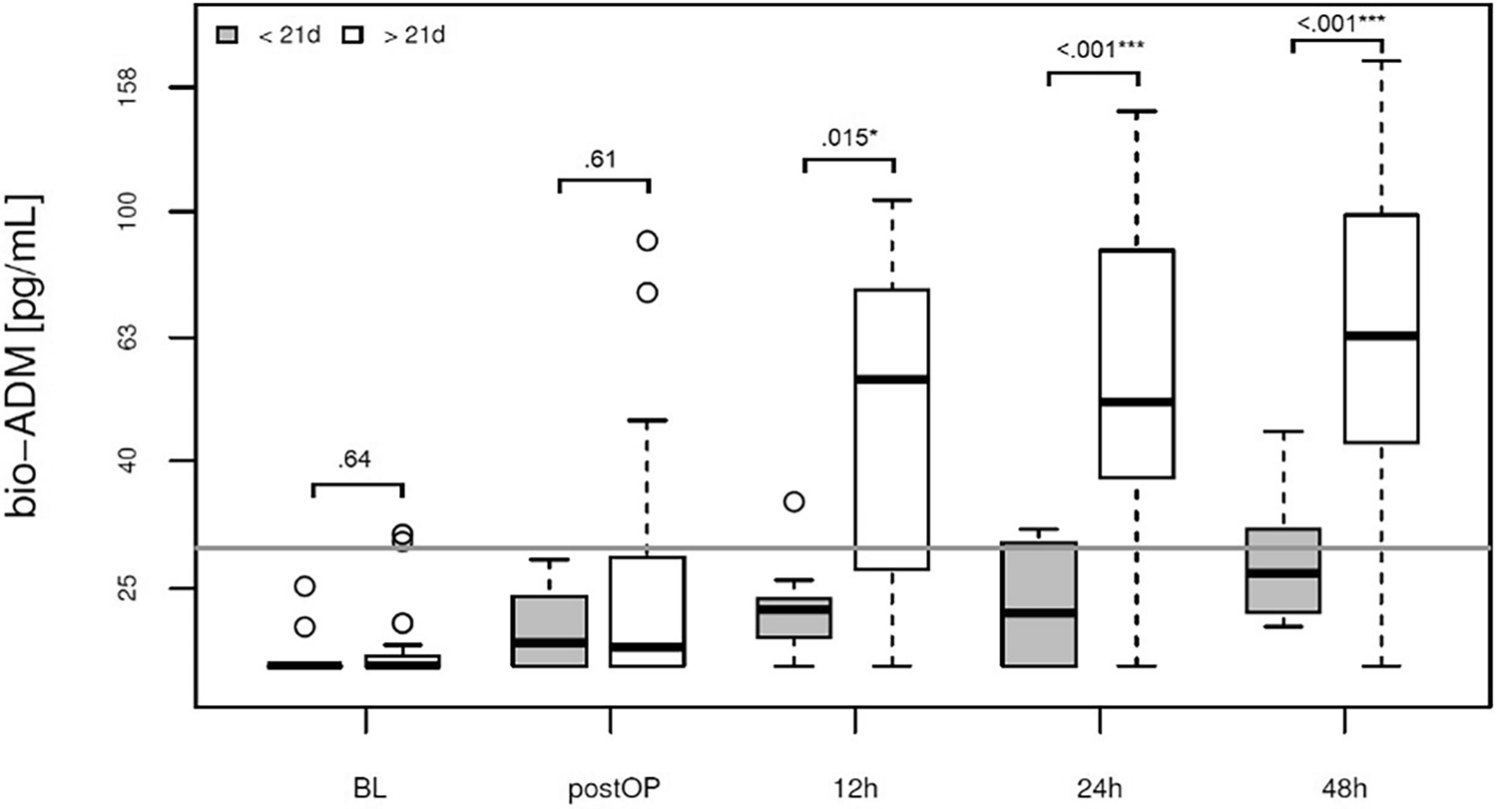
Association of prolonged ventilation and bio-ADM concentration in plasma at baseline, directly postoperatively and at 12, 24 and 48h after surgery (boxplot). White: continuous mechanical ventilation for more than 21 days (n = 23), Grey: continuous mechanical ventilation for less than 21 days (n = 11).

**Figure 2:**
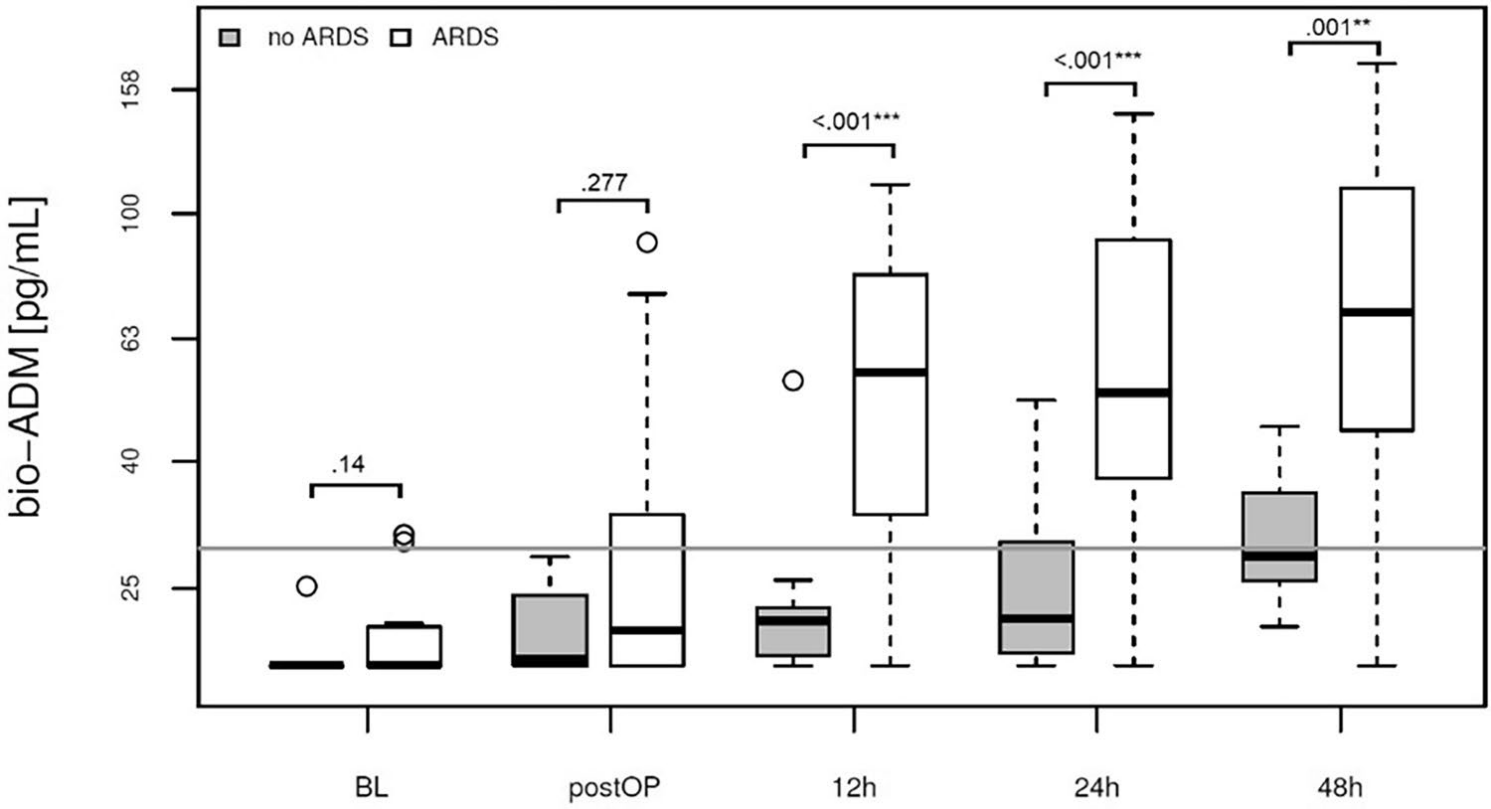
Association of ARDS incidence and bio-ADM concentration in plasma at baseline, directly postoperatively and at 12, 24 and 48h after surgery (boxplot). White: ARDS stages 1–3 (n = 22), Grey: no ARDS (n = 12). ARDS: acute respiratory distress syndrome.

Starting at the 12-h post-surgery time point, patients who required continuous mechanical ventilation for over 21 days exhibited persistent and significantly elevated bio-ADM levels at all subsequent measurements (Fig. 1, Table 2). Additionally, an association was noted between bio-ADM levels and the severity of acute respiratory distress syndrome (ARDS), with patients diagnosed with severe ARDS (stage 3) demonstrating the most pronounced elevation of the biomarker, commencing 12 h after surgery (Fig. 2, Supplementary Fig. 1). In the ROC-Curve analysis starting from the 12h time point, bio-ADM levels exhibited an area under the curve (AUC) of 0.862. The optimal cut off based on the Youden Index is 32.4 pg/mL and corresponded to a Sensitivity of 82% and Specificity of 92% (see supplementary Fig. 2 and Supplementary Table 1 for results at 24h and 48h). The diagnosis of ARDS according to clinical criteria of the Berlin definition was made in 6.4 ± 5.3 days after surgery. CRP levels at baseline, 24h and 48h after surgery did not correlate with the later onset of ARDS (Supplementary Fig. 3).

**Table 2:** Postoperative outcomes and their association with mortality, sepsis, onset of ARDS and bio-ADM levels at 24 h after surgery. The median of bio-ADM at 24 h postoperatively (39.7 pg/mL) was used to dichotomize the continuous variable.

	n	All	Non-fatal	Fatal	*p*-value	No sepsis	Sepsis	*p*-value	No ARDS	ARDS	*p*-value	Bio-ADM, 24 h [0,39.7) pg/mL	Bio-ADM, 24 h (39.7,180] pg/mL	*p*-value
Outcomes														
Death (%)	36	9 (25)	0 (0)	9 (100)	-	4 (18.2)	5 (45.5)	.21	2 (16.7)	7 (31.8)	.58	3 (16.7)	5 (31.2)	.55
Hospital stay (days)—median [IQR]	33	37 [25–59]	38.5 [28–66]	37 [13–52]	.36	36 [21.5–45.75]	66 [42.5–83.5]	.03*	26 [16.75–39]	51 [34–66]	.02*	29 [21–45]	52 [35–66]	.04*
ICU stay (days)—median [IQR]	34	21 [8.5–47]	15 [8–44]	37 [13–52]	.39	13 [6.5–28.5]	48 [34.5–63]	.01*	10 [6–13.25]	44 [27–56]	.001*	12.5 [6.25–18.75]	37 [25.5–51]	.01*
Ventilation time (hours)—median [IQR]	33	347 [12–802]	217.5 [11.75–674.75]	529 [246–1107]	.18	36 [11.25–490.5]	1107 [516–1382.5]	.001*	12 [10.75–49]	634 [432–1209]	.001*	12 [10–158]	797 [516–1158]	.001*
Prolonged ventilation (%)	34	23 (67.6)	15 (62.5)	8 (88.9)	.3	12 (54.5)	11 (100)	.02*	4 (33.3)	19 (90.5)	.001*	7 (41.2)	15 (100)	.001*
Need for catecholamins (days)—median [IQR]	34	8 [3–20.75]	6.5 [2.75–11.25]	13 [8–34]	.21	4.5 [2–9.75]	29 [10–34]	.01*	2.5 [1.75–4.25]	11 [8–33]	.001*	3 [2–6]	16.5 [9.75–33.25]	.001*
Noradrenalin max. dosis [(ug/kg)/min]—median [IQR]	32	.32 [.13-.56]	0.2 [0.07–0.43]	0.63 [0.5–0.96]	.001*	0.2 [0.06–0.45]	0.5 [0.32–0.84]	.01*	0.2 [0.07–0.35]	0.38 [0.19–0.58]	.08	0.12 [0.05–0.23]	0.44 [0.33–0.58]	.001*
Need for catecholamins > = 3 days (%)	35	26 (74.3)	18 (75)	7 (77.8)	1	15 (68.2)	10 (90.9)	.31	6 (50)	19 (90.5)	.03*	10 (58.8)	15 (93.8)	.05
Mass transfusion (%)	34	26 (76.5)	18 (75)	7 (77.8)	1	14 (66.7)	10 (90.9)	.28	7 (63.6)	18 (81.8)	.47	11 (68.8)	14 (82.4)	.61
Platelet concentrate (bags)—median [IQR])	35	5 [3–5.5]	4 [3–5]	8 [5–8]	.01*	4 [3–5]	7 [5–8]	.001*	4 [2.75–5]	5 [4–6.75]	.19	4 [2–5]	5 [4–7]	.06
Packed red blood cells (bags) – median [IQR]	35	18 [11.5–29]	16 [10–28]	27 [24–31]	.1	13.5 [10–22.5]	29 [26.5–37]	.01*	12 [9.75–26.75]	21 [16–29.75]	.13	12 [7–18]	28 [16–31]	.001*
FFP—median [IQR]	35	26 [15.5–34]	22 [14–26]	41 [26–45]	.01*	23 [14.25–29.25]	26 [23–36]	.23	23.5 [15–33]	26 [16.25–30.75]	.87	22 [12–27]	26 [24–36]	.08
PCC- median [IQR]	35	3 [.5–4.5]	2 [1–4]	3 [0–6]	.57	2 [0–3.75]	3 [2.5–6]	.04*	2 [0.75–4.25]	3 [0.25–4]	.96	2 [0–4]	3 [2–4]	.29
Fibrinogen (g)—median [IQR]	35	6 [3–8]	4 [4–6]	6 [0–12]	.33	4 [0.5–6]	8 [6–11]	.01*	4 [3.5–6.5]	6 [1–8]	.56	4 [2–8]	6 [4–8]	.73
Visceral Malperfusion (%)	36	8 (22.2)	4 (15.4)	4 (44.4)	.18	3 (13.6)	4 (36.4)	.29	2 (16.7)	6 (27.3)	.78	3 (16.7)	5 (29.4)	.62
Pneumonia (%)	35	26 (74.3)	18 (69.2)	8 (88.9)	.47	13 (59.1)	11 (100)	.04*	4 (33.3)	21 (95.5)	.001*	9 (50)	16 (100)	.001*
Renal replacement therapy (%)	35	23 (65.7)	13 (52)	9 (100)	.03*	10 (45.5)	11 (100)	.01*	4 (33.3)	18 (81.8)	.01*	7 (41.2)	15 (88.2)	.01*
ARDS (%)	34	22 (64.7)	15 (60)	7 (77.8)	.58	11 (50)	10 (90.9)	.06	0 (0)	22 (100 =	-	6 (35.3)	15 (93.8)	.001*
Sepsis (%)	33	11 (33.3)	6 (25)	5 (55.6)	.21	0(0)	11 (100)	-	1 (8.3)	10 (47.6)	.06	3 (17.6)	8 (53.3)	.08
Liver failure (%)	33	7 (21.2)	0 (0)	7 (77.8)	.001*	3 (13.6)	4 (36.4)	.29	2 (16.7)	5 (23.8)	.97	2 (11.8)	4 (26.7)	.53
Multiorgan dysfunction syndrome (%)	34	9 (26.5)	2 (8)	7 (77.8)	.001*	3 (13.6)	5 (45.5)	.11	2 (16.7)	7 (31.8)	.58	2 (11.8)	6 (37.5)	.19
Atrial fibrillation (%)	29	8 (27.6)	6 (28.6)	2 (33.3)	1	3 (17.6)	5 (50)	.18	0 (0)	8 (50)	.02*	3 (20)	5 (38.5)	.51

ICU, intensive care unit; FFP, fresh frozen plasma; PCC, Prothrombin complex concentrate; ARDS, acute respiratory distress syndrome.

### Elevated bio-ADM levels are associated with catecholamine therapy, postoperative atrial fibrillation, and mortality.

**Figure 3:**
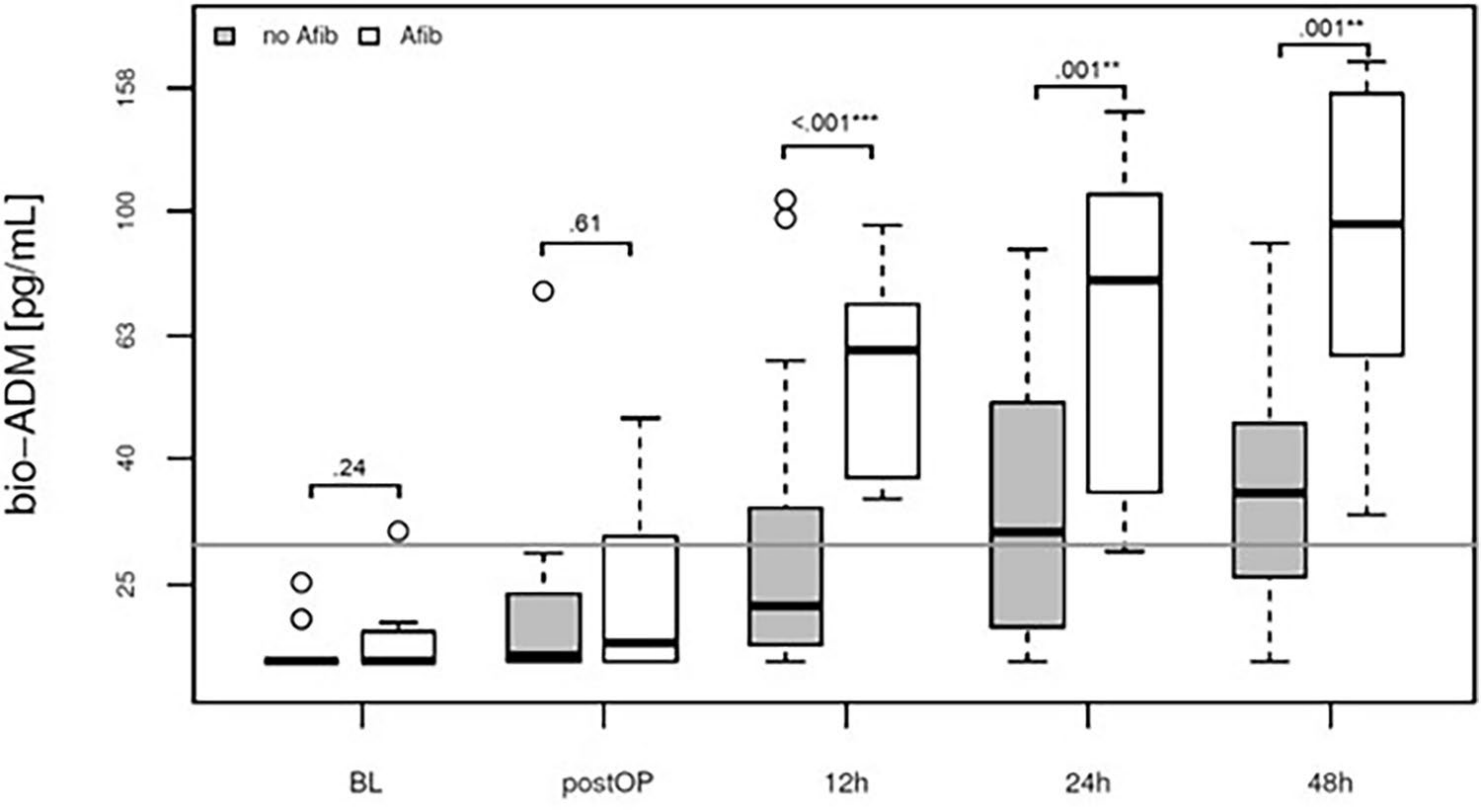
Association of atrial fibrillation and bio-ADM concentration in plasma at baseline, directly postoperatively and at 12, 24 and 48h after surgery (boxplot). White: atrial fibrillation (n = 8), Grey: no atrial fibrillation (n = 21). Afib: atrial fibrilation.

**Figure 4:**
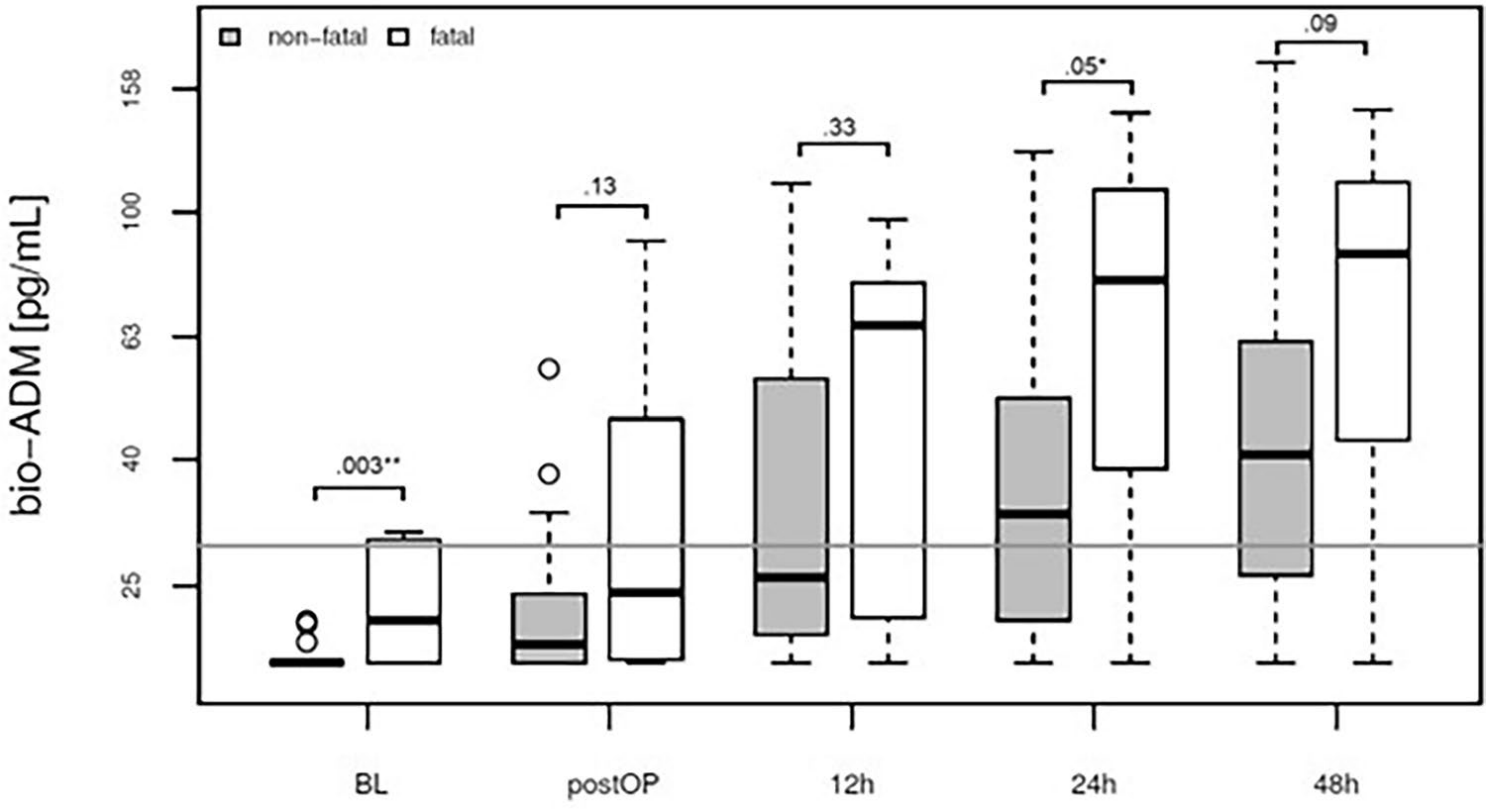
Association of fatal outcomes and bio-ADM concentration in plasma at baseline, directly postoperatively and at 12, 24 and 48h after surgery (boxplot). White: fatal outcome (n = 9), Grey: non-fatal outcome (n = 27).

Patients with elevated bio-ADM levels at the 12-h time point after surgery demonstrated a significantly higher incidence of atrial fibrillation compared to those with lower levels (Fig. 3). Moreover, our study revealed a significant association between sustained elevation of bio-ADM levels at the 24-h mark after surgery and fatal outcomes (Fig. 4) and patients with elevated bio-ADM levels required a significantly prolonged administration of catecholamines to maintain normotension, as demonstrated in Supplementary Fig. 4. CRP levels at baseline and at 24 and 48h after surgery did not correlate with mortality (Supplementary Fig. 5).

### Sepsis and multi-organ failure associated with bio-ADM levels after the 12th postoperative hour

**Figure 5:**
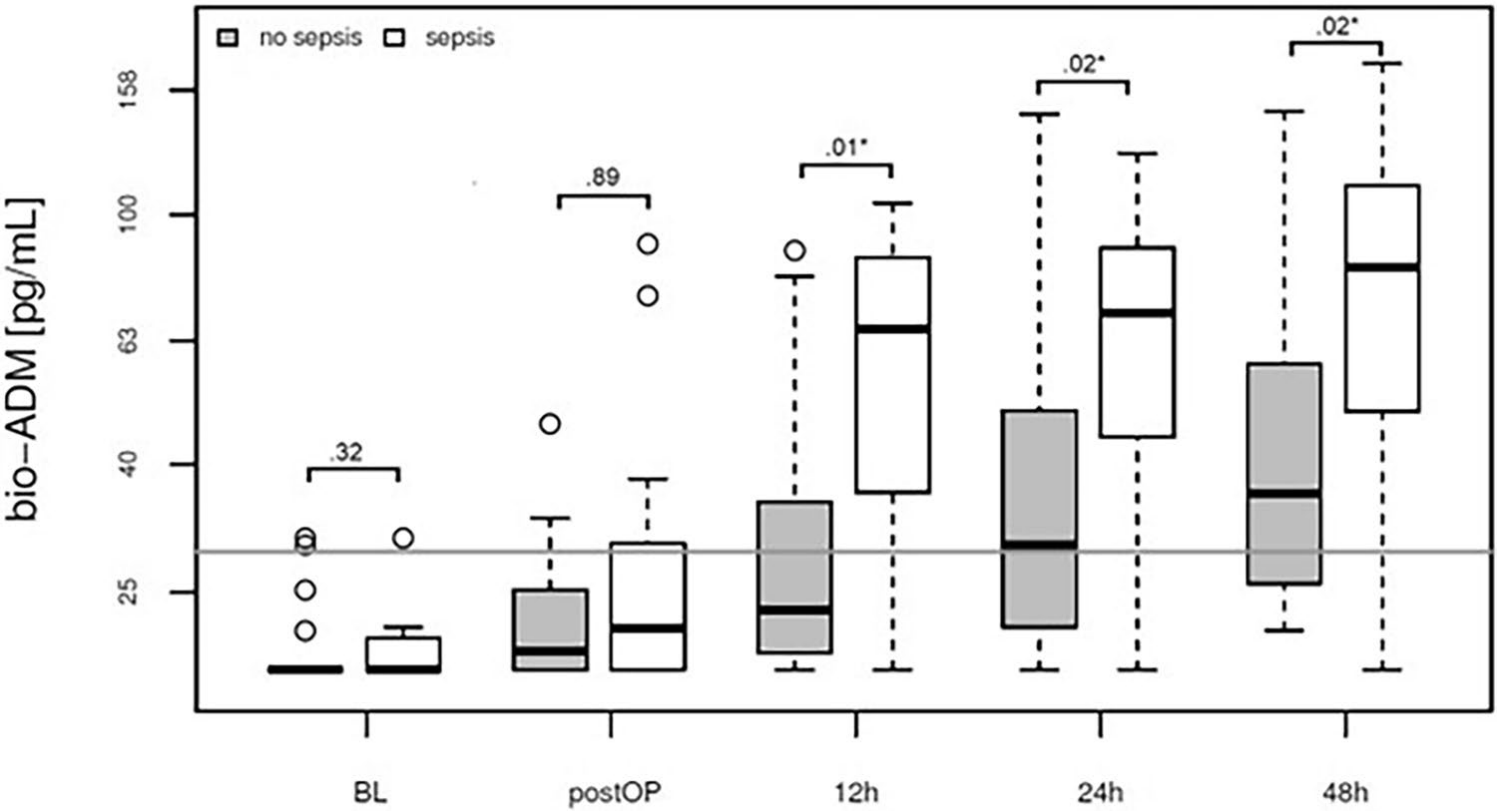
Association of sepsis and bio-ADM concentration in plasma at baseline, directly postoperatively and at 12, 24 and 48h after surgery (boxplot). White: sepsis (n = 11), Grey: no sepsis (n = 22).

From the 12-h mark following surgery, there was a clear increase in bio-ADM among patients who developed sepsis (Table 2, Fig. 5). The diagnosis of sepsis was made on average 12.5 ± 9.4 days after surgery and CRP levels at baseline, at 24h and 48h after surgery did not correlate with its onset (Supplementary Fig. 6). Pneumonia was diagnosed in all patients with sepsis and was identified as the leading cause for sepsis. Furthermore, a direct association emerged between bio-ADM levels and the occurrence of multi-organ failure (MOF). Notably, patients experiencing MOF displayed the most relevant increase in the biomarker, directly postoperatively and at the 24-h time point (Supplementary Fig. 7).

## Discussion

In this retrospective, observational trial, we report a significant association between bio-ADM levels from 12 to 48 h after open TAAA surgery and the incidence of respiratory instability as well as fatal outcomes and SIRS-related morbidity. The biomarker was associated with the later incidence of ARDS, particularly ARDS stage 3 and prolonged ventilation, as well as the onset of sepsis and multi-organ dysfunction syndrome. Patients with persistently elevated postoperative bio-ADM levels required significantly longer duration of catecholamine therapy during their ICU stay to maintain a stable circulatory situation.

In the current era of thoracoabdominal aortic surgery, most cases are treated endovascularly. However, open surgery remains the treatment of choice for certain patient groups—particularly young patients with connective tissue diseases and patients with anatomies not suitable for endovascular procedures, while this subgroup of patients is constantly getting smaller as endovascular techniques evolve^29^. Patients undergoing open TAAA reconstructions are prone to postoperative hemodynamic instability and organ dysfunction^30^. This is attributed to a multifactorial interplay of cellular processes and cytokine-mediated systemic inflammation triggered during the procedure and the early postoperative phase and which has been linked to organ dysfunction^31,32^ and susceptibility to infections^9^. The lungs are often the centrepiece of this cascade^9^ and in a previous study of our group, we observed, that, indeed, the intraoperative cytokine removal through the Cytosorb®-system was associated with reduced invasive ventilation times and incidence of severe ARDS^33^. In line with these findings, we assume that patients who were later diagnosed with severe ARDS and required prolonged ventilation, displayed compromised endothelial integrity and capillary leakage as possible underlying pathomechanism during the first postoperative hours, as indicated by an increase in bio-ADM. Early detection of the patients at risk for ARDS remains of paramount importance, since it is associated with relevant mortality rates of 30–40%^34^. Given the multitude of stimuli and their heterogeneity, that may contribute to the onset of ARDS, the need of biomarkers as an extension of clinical scores for the prediction of ARDS-related mortality has been highlighted in the literature^35,36^. Johnsson et al. recently reported a significant elevation of bio-ADM levels at the time of admission in ICU in patients that were diagnosed with ARDS within 72 h after admission^37^. Building upon the findings of this study, which included a large cohort of patients with a wide range of conditions, we investigated the dynamics of bio-ADM levels during the first 48 h following open TAAA repair. In this homogeneous cohort, the biomarker was significantly associated with the later onset of ARDS, which was diagnosed at a mean of 6.4 days after surgery. This association was observed as early as 12 h after surgery, and using a cut-off of 32.4 pg/mL, bio-ADM could identify the patients at risk for ARDS with a sensitivity of 82% and specificity of 92%, potentially providing a valuable advantage for their management during the early postoperative phase.

Capillary leakage and poor systemic vascular resistance naturally affect the patient’s hemodynamic stability and require vasopressors to maintain stable systemic blood pressure and organ perfusion^38^. The underlying mechanisms for vasoplegia in patients undergoing open TAAA repair, start in the operating room with the connection of extracorporeal circulation. Exposure of the blood to the surface of the extracorporeal circulation circuit induces the release of inflammatory mediators, such as interleukin-1 (IL-1), interleukin-6 (IL-6), and tumor necrosis factor-alpha (TNFa), which stimulate the production of nitric oxide, resulting in vasodilation^39^. This cascade can be further escalated through the cytokine surge caused by the surgical trauma and aortic cross-clamping^40^. Furthermore, visceral ischemia–reperfusion-injury perioperatively may induce cellular stress and trigger an elevated cytokine response^8,41^. In the cohort studied, postoperative visceral malperfusion did not show a significant correlation with the onset of ARDS, sepsis, or mortality, nor did it significantly influence bio-ADM levels at 24 h postoperatively. This suggests that the mechanisms influencing these clinical outcomes may operate independently of the vascular and inflammatory responses typically associated with visceral malperfusion.

Increased bio-ADM levels have been found to offer valuable guidance in risk stratification in hemodynamically impaired patients^21,42^. The diagnostic value of bio-ADM in patients undergoing cardiac surgery was recently reported by Hill et al., who observed an association between bio-ADM levels and the need for vasopressors, indicating compromised circulation in this patient population^43^. Our findings confirm the observations of these previous trials and expand them to the field of open TAAA surgery: bio-ADM was directly associated to catecholamine therapy at all postoperative time points, starting from the 12-h mark and could detect the patients with a fatal outcome as soon as 24 h postoperatively.

Furthermore, persistent elevation of bio-ADM after surgery was significantly associated with the onset of postoperative atrial fibrillation (POAF) during their ICU stay, a condition in which inflammation seems to play a key role^44^. In our cohort, bio-ADM levels were significantly associated with the onset of POAF as soon as 12 h postoperatively, as the biomarker identified the patients at risk for inflammatory events, which may subsequently contribute to cardiac arrhythmia.

The diagnostic and prognostic value of bio-ADM in sepsis and septic shock is well documented in literature^13,16,18–20,45–48^. Patients with SIRS are susceptible to infections, leading to the clinical manifestation of sepsis, which in turn is a relevant risk factor for multiple organ failure^28^. Early detection of patients at risk is crucial to avoid sepsis-related complications and the related early and late mortality rates^49^. In our cohort, starting at the 12-h mark bio-ADM demonstrated its utility in this regard, showing a significant association with the later onset of sepsis. Consequently, we observed a relevant, positive association of bio-ADM levels and occurrence of MOF from the 24-h time point. However, in the context of sepsis, the role of ADM is not limited only to the regulation of vascular tone and the endothelial barrier, but also expands to immunoregulation and antimicrobial protection^13^. In vitro and in vivo animal experiments have shown that ADM has a direct cAMP-mediated anti-inflammatory effect on macrophages^50^ and provides protection from infections through inhibition of bacterial growth^51^. This notion suggests, besides the diagnostic properties of bio-ADM, also a possible therapeutic perspective in patients with sepsis^52^. Preliminary studies on the non-neutralising ADM-binding antibody adrecizumab show promising results in animal models in reducing vascular leakage, organ dysfunction and need for catecholamines to maintain stable circulatory conditions^53^. A recently conducted phase 2a bio-ADM-guided trial has shown the safety and tolerability of adrecizumab in septic shock patients^54^. Given the increased risk for sepsis and septic shock in patients undergoing open TAAA repairs, bio-ADM might be an important element of the personalized, precision medicine of the future.

In summary, monitoring bio-ADM levels in the early postoperative phase provides critical insight into subclinical cytokine-driven capillary impairment, which predisposes patients to the subsequent onset of ARDS. Additionally, this capillary leakage serves as a shared pathophysiological route for both ARDS and sepsis. The early identification of at-risk patients is crucial for mitigating postoperative mortality and morbidity. Integrating bio-ADM measurements into standard clinical practice could offer clinicians a valuable tool for directing therapeutic interventions and enhancing overall patient outcomes.

It is important to acknowledge the relevant limitations of this retrospective, observational study in order to properly interpret the reported results. Firstly, the enrolment of only a small number of patients was possible due to the increasingly falling number of yearly-performed procedures for open TAAA repairs. The small sample size may limit the generalizability of the reported results to larger populations. Furthermore, it is important to note that this study specifically concentrated on analysing the dynamic fluctuations in bio-ADM levels during the early postoperative phase, aiming to simulate a point-of-care approach in routine clinical practice. Bio-ADM was chosen as the biomarker of interest based on its proven role in vascular integrity and endothelial function, which are critical in postoperative complications. The investigation did not explore alterations in other proinflammatory factors and cytokines that are recognized to contribute to SIRS and although elevated bio-ADM levels might indicate endothelial damage or leakage, this mechanism was not verified through specific markers of endothelial integrity. Future studies could benefit from a multi-marker approach to more fully delineate the complex interplay of inflammatory responses in postoperative patients. Nevertheless, the homogeneity of the cohort under examination and the standardized operative protocols implemented in both centres provide support for the proposed pathways and conclusions. To confirm the diagnostic and potentially therapeutic significance of this biomarker in the realm of open aortic surgery, future research involving larger-scale, multicentre trials is warranted.

## Conclusion

Elevated bio-ADM levels within the first 48 h after open TAAA repair were significantly related to increased incidence of postoperative respiratory failure—particularly ARDS and prolonged ventilation—atrial fibrillation, sepsis, and multiple organ failure. Considering the hemodynamic instability of these patients due to systemic inflammation, there is a relevant risk for a fatal outcome—which was significantly associated with the biomarker as soon as 24 h after surgery.

### Supplementary Information

Supplementary Information.

## Data Availability

The raw data supporting the conclusions of this article will be made available by the corresponding author (Panagiotis Doukas), without undue reservation.
