# Supplementary material for: Postoperative bioactive adrenomedullin is associated with the onset of ARDS and adverse outcomes in patients undergoing open thoracoabdominal aortic surgery
Source: Sci Rep. 2024 Jun 4;14:12795. doi: 10.1038/s41598-024-63412-1 (PMC11150250; doi:10.1038/s41598-024-63412-1)
Supplement: Supplementary file 1 — Supplementary Information. [file 41598_2024_63412_MOESM1_ESM.pdf]

Supplement:

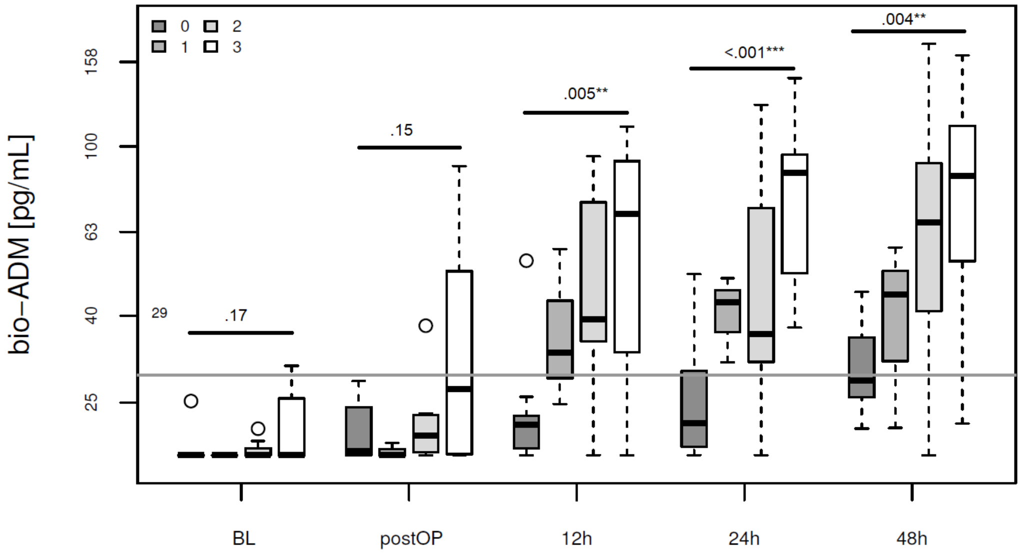

Supplementary Figure 1: Dynamic of bio-ADM concentration in plasma at baseline, directly postoperatively and at 12, 24 and 48h after surgery and its association with ARDS stages (boxplot).

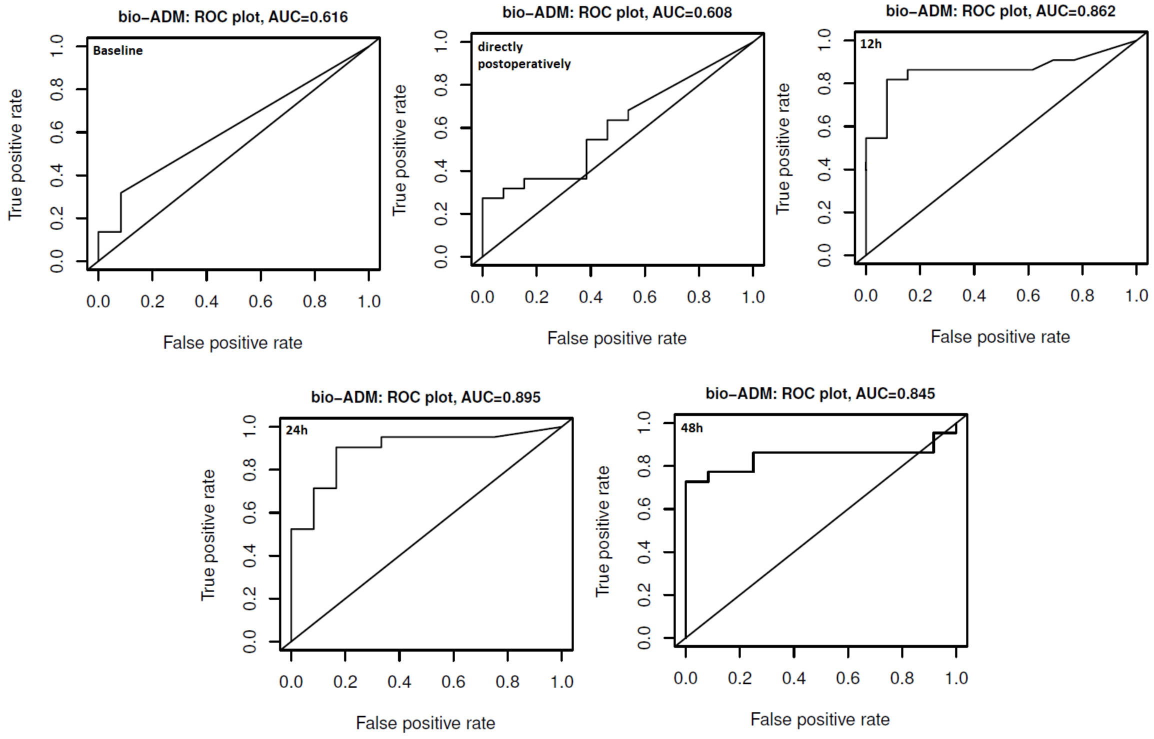

Supplementary Figure 2: Receiver-Operating-Characteristic curves for bio-ADM at baseline, directly postoperatively and at 12, 24 and 48h after surgery and the onset of ARDS.

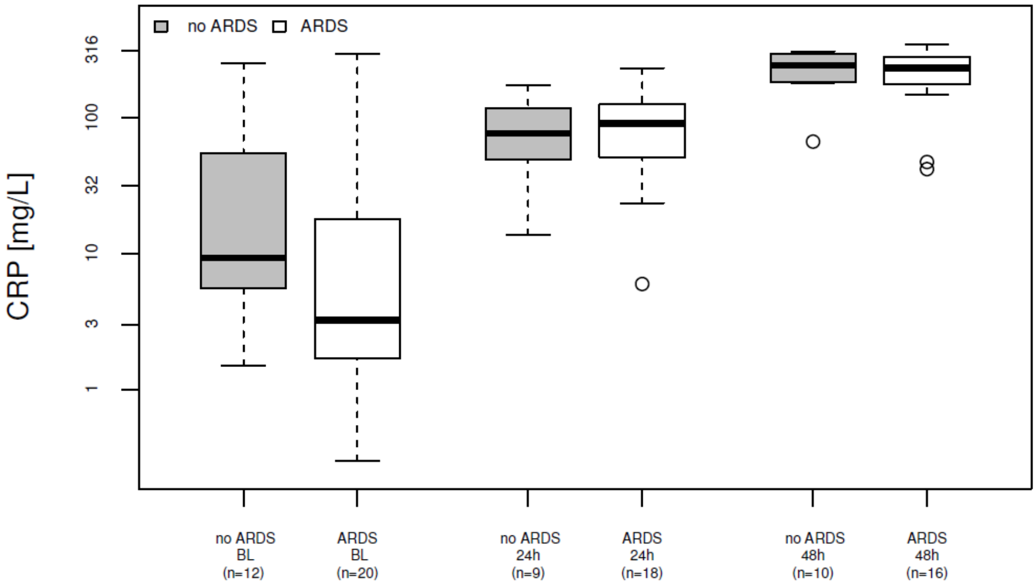

Supplementary Figure 3: Dynamic of CRP concentration in plasma at baseline, at 24 and 48h after surgery and its association with ARDS stages (boxplot). CRP: C-reactive protein.

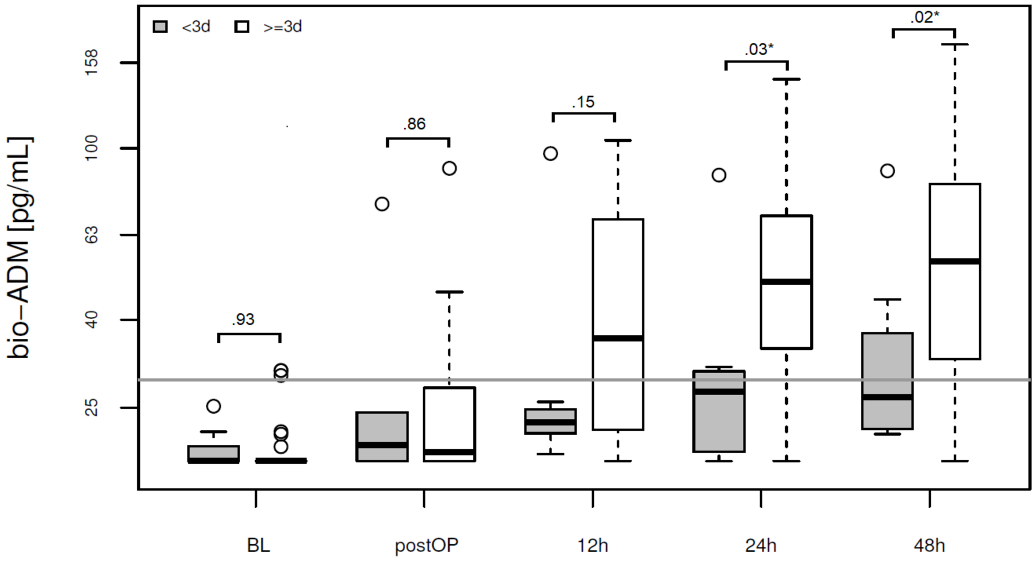

Supplementary Figure 4: Dynamic of bio-ADM concentration in plasma at baseline, directly postoperatively and at 12, 24 and 48h after surgery (boxplot). White: catecholamine therapy for more than 3 days, Grey: catecholamine therapy for less than 3 days

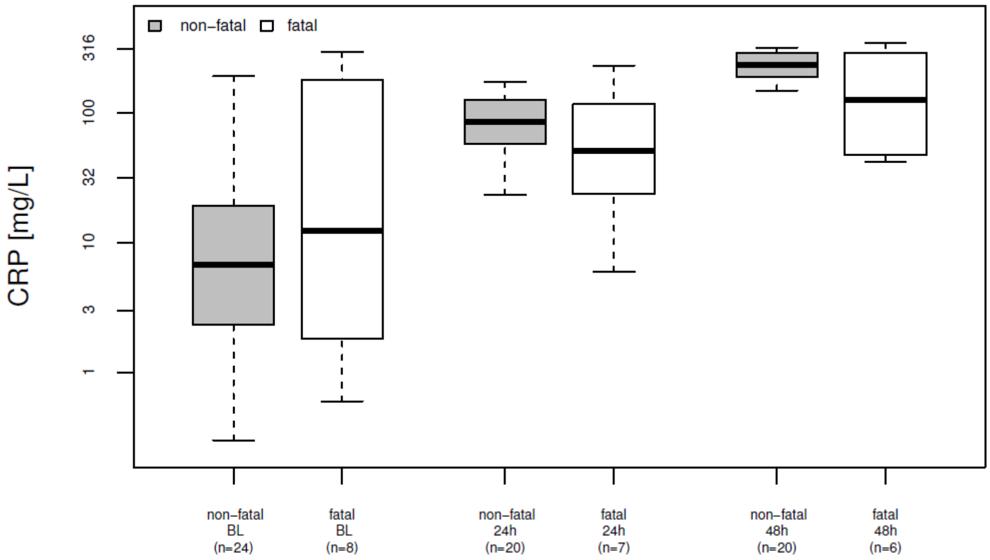

Supplementary Figure 5: Dynamic of CRP concentration in plasma at baseline, at 24 and 48h after surgery and its association with mortality (boxplot). CRP: C-reactive protein.

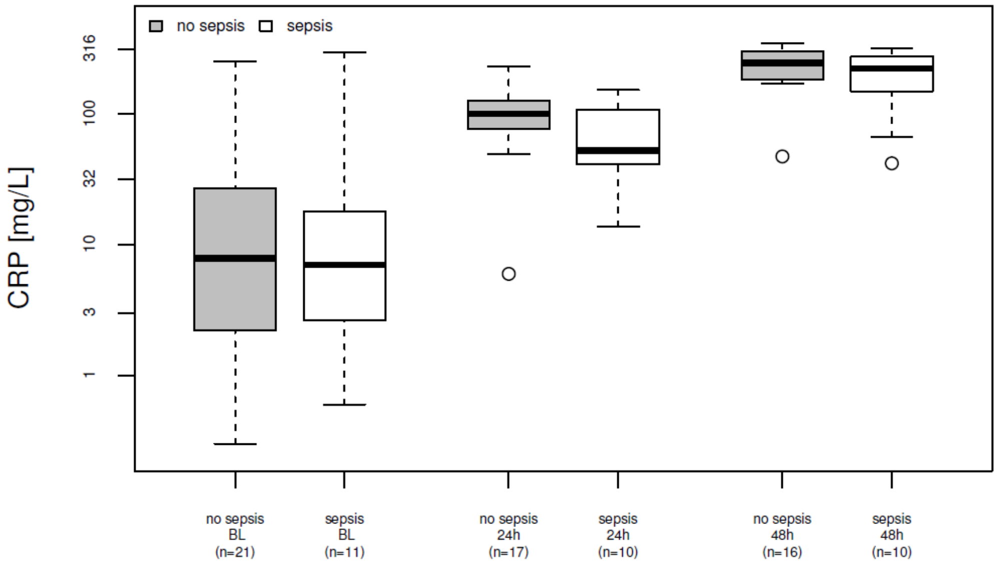

Supplementary Figure 6: Dynamic of CRP concentration in plasma at baseline, at 24 and 48h after surgery and its association with sepsis (boxplot). CRP: C-reactive protein.

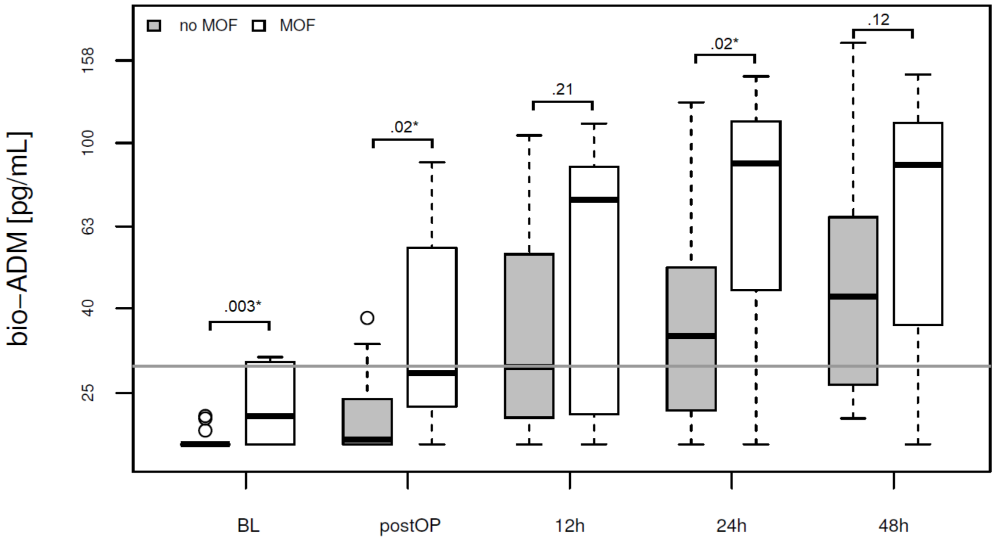

Supplementary Figure 7: Dynamic of bio-ADM concentration in plasma at baseline, directly postoperatively and at 12, 24 and 48h after surgery (boxplot). White: multi-organ failure, Grey: no multi-organ failure

Supplementary Table 1: Receiver-operating-characteristic curve analysis for bio-ADM levels at 12h, 24h and 48 h after surgery and the onset of ARDS. AUC: Area under the curve

Timepoint	*p-value*	*c-index [95-CI]*	*Cut-off (Youden Index)*	*Sensitivity*	*Specificity*
12 h	<.001	.862 [.736 - .988]	32.4 pg/mL	82%	92%
24 h	<.001	.895 [.784 – 1.006]	31.1 pg/mL	91%	83%
48 h	<.001	.845 [.706 - .983]	45.6 pg/mL	73%	100%
